# Rapamycin inhibits the secretory phenotype of senescent cells by a Nrf2‐independent mechanism

**DOI:** 10.1111/acel.12587

**Published:** 2017-03-31

**Authors:** Rong Wang, Zhen Yu, Bharath Sunchu, James Shoaf, Ivana Dang, Stephanie Zhao, Kelsey Caples, Lynda Bradley, Laura M. Beaver, Emily Ho, Christiane V. Löhr, Viviana I. Perez

**Affiliations:** ^1^ Linus Pauling InstituteOregon State UniversityCorvallisOR97331USA; ^2^ Department of Biochemistry and BiophysicsOregon State UniversityCorvallisOR97331USA; ^3^ School of Biological & Population Health SciencesOregon State UniversityCorvallisOR97331USA; ^4^ Department of Biomedical SciencesOregon State UniversityCorvallisOR97331USA

**Keywords:** β‐gal, cell senescence, Nrf2, rapamycin, SASP

## Abstract

Senescent cells contribute to age‐related pathology and loss of function, and their selective removal improves physiological function and extends longevity. Rapamycin, an inhibitor of mTOR, inhibits cell senescence *in vitro* and increases longevity in several species. Nrf2 levels have been shown to decrease with aging and silencing Nrf2 gene induces premature senescence. Therefore, we explored whether Nrf2 is involved in the mechanism by which rapamycin delays cell senescence. In wild‐type (WT) mouse fibroblasts, rapamycin increased the levels of Nrf2, and this correlates with the activation of autophagy and a reduction in the induction of cell senescence, as measured by SA‐β‐galactosidase (β‐gal) staining, senescence‐associated secretory phenotype (SASP), and p16 and p21 molecular markers. In Nrf2KO fibroblasts, however, rapamycin still decreased β‐gal staining and the SASP, but rapamycin did not activate the autophagy pathway or decrease p16 and p21 levels. These observations were further confirmed *in vivo* using Nrf2KO mice, where rapamycin treatment led to a decrease in β‐gal staining and pro‐inflammatory cytokines in serum and fat tissue; however, p16 levels were not significantly decreased in fat tissue. Consistent with literature demonstrating that the Stat3 pathway is linked to the production of SASP, we found that rapamycin decreased activation of the Stat3 pathway in cells or tissue samples from both WT and Nrf2KO mice. Our data thus suggest that cell senescence is a complex process that involves at least two arms, and rapamycin uses Nrf2 to regulate cell cycle arrest, but not the production of SASP.

## Introduction

Cellular senescence, defined as a cell cycle arrest state where cells cannot divide but are still viable, has been implicated in a number of pathologies including age‐related diseases. It is believed that this is primarily due to the secretion of inflammatory molecules that alter the ability of adjacent cells to properly function (Campisi & d'Adda di Fagagna, 2007).

Although cellular senescence can be triggered by multiple stimuli, that is, telomere shortening, DNA damage, and mitogenic oncogenes, there are common markers used to identify senescent cells in addition to growth arrest. These include increased p16 and p21 gene expression, increased senescence‐associated β‐galactosidase staining (β‐gal), and the presence of secretory inflammatory cytokines and other factors known as SASP (senescence‐associated secretory phenotype; Rodier & Campisi, 2011). Although it is known that none of these markers is unique to the senescence state (Collado *et al*., 2007), concurrent occurrence of elevated levels of p16 protein together with β‐gal staining and SASP has been widely used as reliable markers of cell senescence.

Rapamycin, an mTOR inhibitor, improves health in several animal models and inhibits cellular senescence in multiple cell types including fibroblasts. For example, rapamycin delays entry into senescence in cells treated with chemical stress (doxorubicin, sodium butyrate, hydrogen peroxide), ionizing radiation, overexpression of p21/waf, mitochondrial stress, or by replicative lifespan (Demidenko *et al*., 2009; Cao *et al*., 2011; Iglesias‐Bartolome *et al*., 2012). Furthermore, Van Deursen's group demonstrated the importance of senescent cells *in vivo* in aging and healthspan. In their studies, they showed that removal of senescent cells promotes normal tissue function, delays the onset of age‐related pathology, and also attenuates the progression of age‐related disorders already established when this approach is applied late in life (Baker *et al*., 2011, 2016). While Van Deursen's group used a genetic manipulation to eliminate senescent cells, more recently Kirkland's group used a pharmacological approach to attain the same result. Using these so‐called senolytics, they showed that partial removal of senescent cells leads to decreased age‐related phenotypes in wild‐type C57BL/6 mice (Zhu *et al*., 2016).

Nrf2 is a pro‐longevity signaling pathway (Harman, 2006) and also a key mediator of the beneficial effects of calorie restriction. In both *C. elegans* and *Drosophila*, Nrf2 activation induces a significant increase in longevity (Sykiotis & Bohmann, 2008; Tullet *et al*., 2008), while in *C. elegans*, drugs believed to be ‘calorie restriction mimetics’ (resveratrol, metformin, and rapamycin) activate the Nrf2 signaling pathway as part of their mechanism of lifespan extension (Chen *et al*., 2005; Kode *et al*., 2008; Onken & Driscoll, 2010).

Studies have found an inverse correlation between aging and Nrf2 expression, both in mouse tissues and *in vitro* during replicative senescence (Shih & Yen, 2007; Duan *et al*., 2009; Kapeta *et al*., 2010). For example, a decrease in both Nrf2 protein and mRNA levels (~65% and 45%, respectively) was found in senescent fibroblasts relative to their presenescent counterparts, and expression of several target genes decreased as well. Similarly, inhibition of Nrf2 by caveolin‐1 promotes stress‐induced premature senescence (Volonte *et al*., 2013). Furthermore, silencing the Nrf2 pathway in human embryonic fibroblasts leads to premature senescence (Kapeta *et al*., 2010). Furthermore, studies by Jodar *et al*. (2011) showed that Nrf2^−/−^ MEFs were prone to immortalization but also showed a short lifespan.

In this study, we used two models of cellular senescence, stress‐induced premature senescence (SIPS) in mouse embryonic fibroblasts (MEFs), and replicative senescence in human WI38 cells. We found that rapamycin reduced senescence markers such as p16 and p21, which were dependent on Nrf2, and correlate with an inhibition of DNA damage indicated by pH2X marker. However, the inhibition of β‐gal staining and SASP was independent of Nrf2 pathway. These results were further corroborated *in vivo* using the Nrf2KO mouse, where rapamycin treatment led to a decrease in SASP and decreased β‐gal staining in fat tissue, but did not decrease the levels of p16 protein. Taken together, our data support recent studies in the field where rapamycin suppressed SASP independently from the effect on cell cycle arrest. Therefore, different molecular aspects of cell senescence are regulated by either Nrf2‐dependent or Nrf2‐independent mechanisms.

## Results

### Rapamycin activates the Nrf2 pathway and inhibits hydrogen peroxide (H_2_O_2_)‐stress‐induced premature senescence (SIPS)

**Figure 1:**
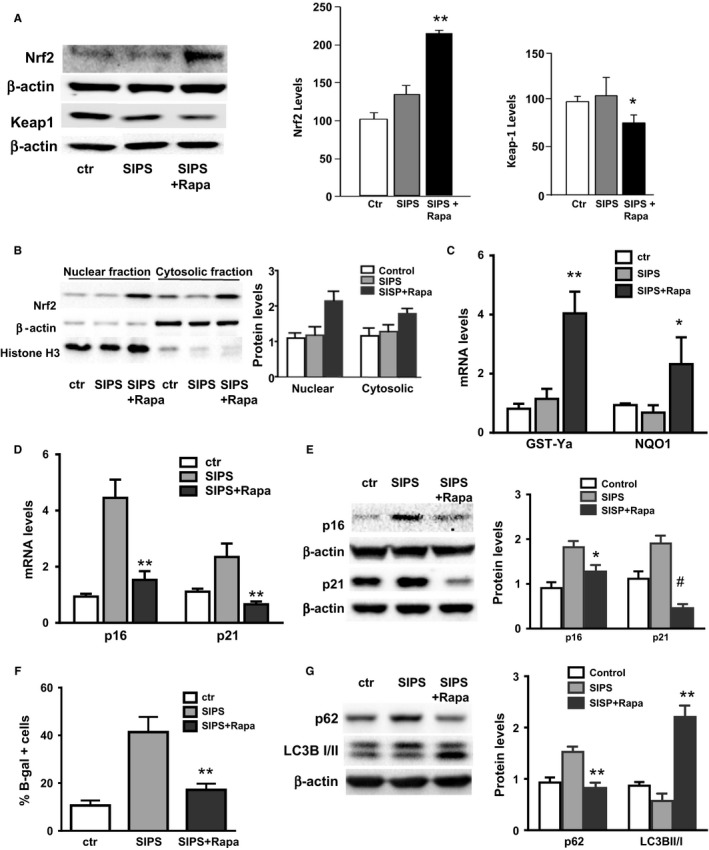
Rapamycin activates Nrf2 pathway and prevents hydrogen peroxide induced SIPS in mouse skin fibroblasts. Cells pretreated with rapamycin (250 nm) for 24 h were exposed to H_2_O_2_ (150 nm) for 2 h. After washing, cells were post‐treated with rapamycin 250 nm and harvested after 24 h (mRNA), 72 h (Western blot), and 6 days (for β‐gal staining). (A) Representative immunoblotting for Nrf2 and Keap1 protein levels with their respective quantification. (B) Representative Western blot and quantification of Nrf2 levels in the cytosolic and nuclear fractions. (C) Nrf2 downstream target gene GST‐Ya and NQO1 mRNA expression levels by qPCR analysis. (D) p16 and p21 mRNA expression levels measured using qPCR analysis. (E) Protein levels of p16, p21. (F) Number of senescent cells measured by β‐gal staining and (G) protein levels of LC3B‐I/II and p62 by immunoblotting. *N* = 3–4 independent experiments. Statistical significance is indicated at *P < 0.05 **P < 0.01 and ^#^ *P* < 0.001.

Pre‐incubation of mouse skin fibroblasts with rapamycin for 24 h increased the levels of Nrf2 in a dose‐dependent manner (Fig. 1A and Fig. S1, Supporting information), and lowered the levels of Keap1, the cytosolic inhibitor of the Nrf2 pathway (Fig. 1A). Activation of the Nrf2 pathway is further demonstrated by the levels of Nrf2 in the nuclear localization (Fig. 1B) and by the increase in mRNA levels of down target genes such GST‐Ya and NQO1 (Fig. 1C). This effect on the Nrf2 pathway correlates with inhibition of cell senescence induced by 2‐h incubation with H_2_O_2_ (150 nm, SIPS), where our results showed that 24 h of pre‐incubation with rapamycin significantly decreased the levels of p16 and p21 molecular markers (Fig. 1D,E), as well as measured by the number of senescent cells measured by β‐gal staining (Fig. 1D–F). As expected, rapamycin treatment also activated autophagy as measured by decreased levels in p62 and increased LC3B‐I to LC3B‐II interconversion (Fig. 1G).

### The inhibition of p16 and p21 levels and activation of autophagy by rapamycin are Nrf2‐dependent

**Figure 2:**
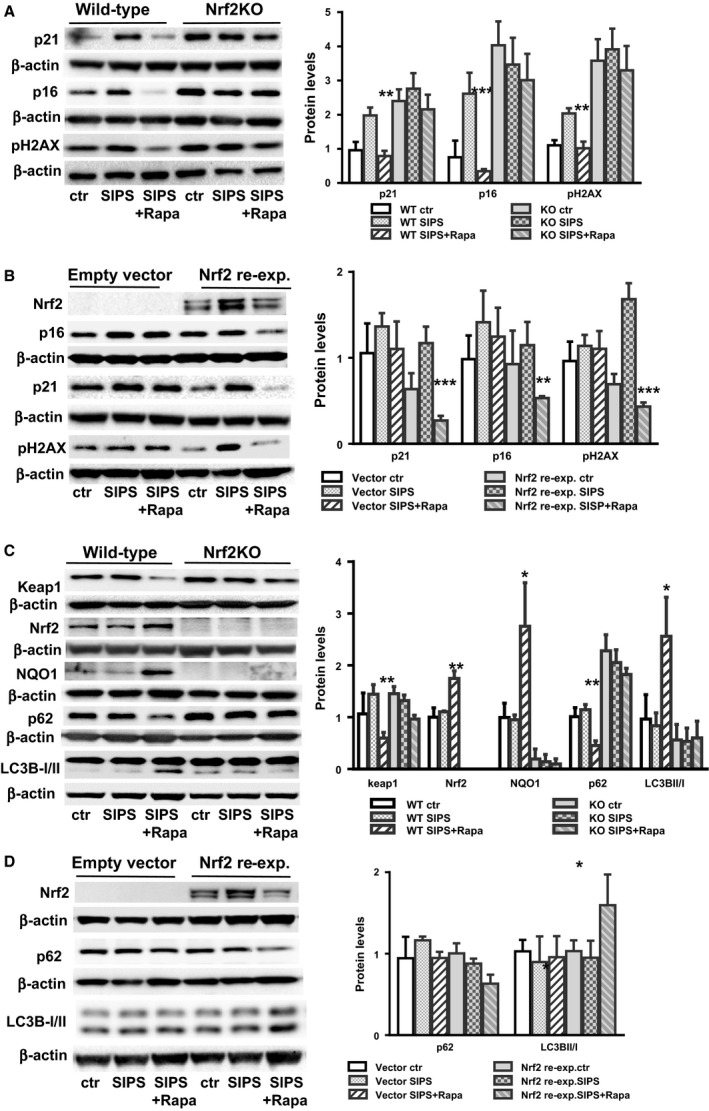
Rapamycin‐induced cell cycle arrest and autophagy activation is dependent on Nrf2. (A). Protein levels for cell cycle arrest markers (p16 and p21) and DNA damage marker pH2AX in wild‐type (WT; left panel) and Nrf2 null cells (Nrf2KO, right panel) in control cells and in response to SIPS or SIPS+ Rapa. (B). Protein levels for p16, p21, and pH2AX in Nrf2KO cells transfected with Nrf2 cDNA (re‐expression) or empty vector. (C) Protein levels for autophagy markers (LC3B‐I/II, p62) in WT (left panel) and Nrf2KO (right panel) in control cells and in response to SIPS or SIPS+ Rapa. (D) Protein levels for LC3B‐I/II and p62 in Nrf2KO cells transfected with Nrf2 cDNA (re‐expression) or empty vector. *N* = 3–4 independent experiments. Statistical significance is indicated at *P < 0.05, **P < 0.01 and ***P < 0.001.

To determine the role of Nrf2 in the inhibition of cell senescence, we compared the effect of rapamycin on WT and Nrf2KO mouse embryonic fibroblasts (MEFs). We observed that using a rapamycin dose of 250 nm led to a significant inhibitory effect on cell proliferation, probably due to a massive inhibition of the mTOR pathway (Fig. S8A, Supporting information). Therefore, after our analysis of cell toxicity and proliferation, we decided to use a lower dose of rapamycin of 25 nm. Similar to what we observed in mouse skin fibroblasts, rapamycin inhibits cellular senescence induced by hydrogen peroxide in WT MEFs, as shown by lower levels of p16 and p21 (Fig. 2A). BrdU incorporation was used to measure the effect on cell proliferation (Fig. S5, Supporting information). We found that this correlates with lower levels of pH2AX, a marker of DNA damage (Fig. 2A), suggesting that rapamycin prevents cell senescence by reducing DNA damage. However, no effects on p21 and p16 levels were observed in Nrf2KO MEFs (Fig. 2A). To confirm that the expression of these markers is Nrf2‐dependent, we re‐expressed Nrf2 in Nrf2KO MEFs by transfection. Compared to control cells transfected with the empty vector, we were able to re‐establish the effect induced by rapamycin on p16, p21 and pH2AX expression (Fig. 2B). Together, our data indicate that Nrf2 is necessary for rapamycin to inhibit expression of the senescent markers p16 and p21. Comparable results were obtained when we used bleomycin (1 μg mL^−1^) instead of H_2_O_2_, suggesting that this may be a general mechanism (Fig. S2, Supporting information). Similarly, we found that rapamycin activates autophagy only in WT cells but not in Nrf2KO MEFs, as measured by the interconversion of LC3I to LC3II and flux analysis (Fig. 2C and Fig. S4, Supporting information), and this effect was re‐established when cells were transfected with the Nrf2 expressing plasmid (Fig. 2D), suggesting that rapamycin activates autophagy in a Nrf2‐dependent manner.

**Figure 3:**
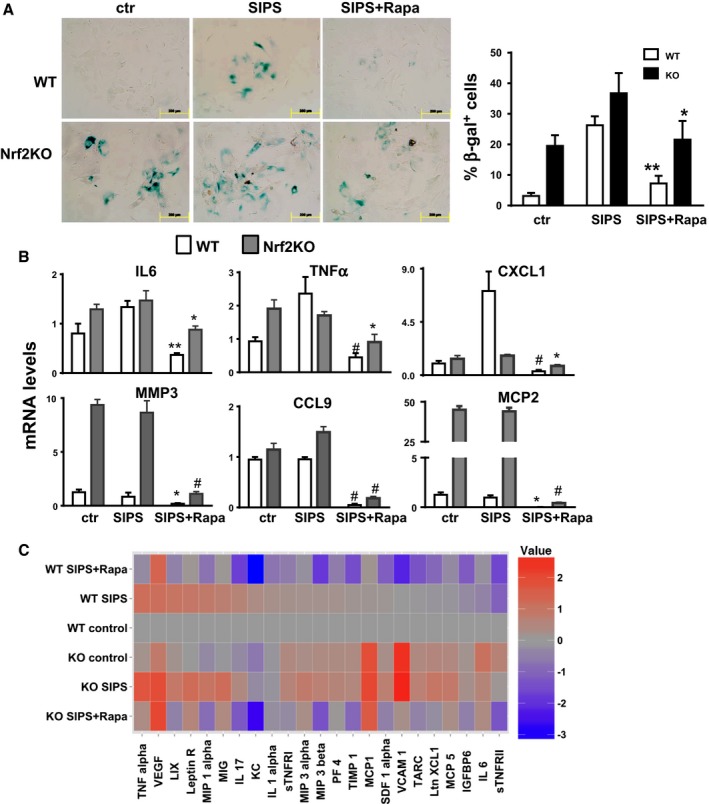
Inhibition of β‐gal staining and SASP by rapamycin is independent of Nrf2. (A) Quantification of β‐gal‐positive cells in WT and Nrf2KO MEF cells in control cells and in response to SIPS or SIPS + Rapa. Open and black bars represent WT and Nrf2KO cells, respectively. (B) mRNA levels of cytokine in WT (gray bars) and Nrf2KO (black bars) MEF cells were measure by qPCR in control cells and in response to SIPS or SIPS + Rapa after 6 days of rapamycin post‐treatment. (C) Cytokine protein levels secreted by WT and Nrf2KO MEF cells in control cells and in response to SIPS or SIPS + Rapa were measured in conditioned media using mouse cytokine antibody array. Data were expressed as relative to WT controls. *N* = 3–4 independent experiments. Statistical significance is indicated at *P < 0.05, **P < 0.01, and ^#^ *P* < 0.001.

We observed that at basal state, MEFs deficient in Nrf2 have higher levels senescent cells than WT cells, as measured by β‐gal staining (Fig. 3A). Surprisingly, we found that rapamycin treatment was able to decrease the number of senescent cells in both WT and Nrf2KO MEFs (Fig. 3A). The same results were observed when we measured the senescence‐associated secretory phenotype (SASP) by mRNA levels (Fig. 3B) or using a mouse cytokine array (Fig. 3C), where rapamycin decreased significantly the SASP in cells and conditioned media from both WT and Nrf2KO MEFs. Together, these data suggest that the inhibition of both β‐gal‐positive cells and the inflammatory phenotype that escorts cellular senescence are Nrf2 independent, while the inhibition of the induction of p16 and p21 is Nrf2 dependent.

### Rapamycin delays the accelerated replicative senescence induced by Nrf2 deficiency in WI38 cells

**Figure 4:**
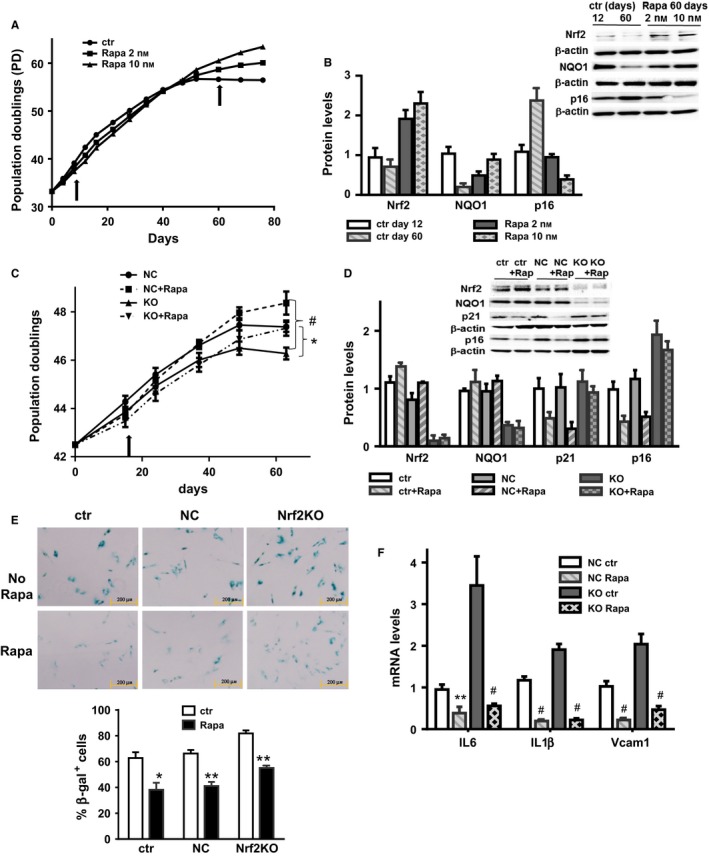
Rapamycin effect on cell cycle arrest in WI38 replicative senescence is Nrf2‐dependent. (A) WI38 cell population doubling levels and (B) protein levels of Nrf2, NQO1, and p16 in cells treated with Rapamycin 2 or 10 nm. (C) WI38 cell population doubling levels and (D) protein levels of Nrf2, NQO1, p21, and p16 in WI38 transfected with Nrf2 Crispr (KO) or using a control gRNA (NC) treated with or without rapamycin 10 nm. Arrows indicate time point of immunoblotting. (E) Representative β‐gal staining picture (and quantitation) in WI38 cells controls, Nrf2KO or NC treated with or without rapamycin (10 nm). (F) mRNA levels for cytokines (interleukin 6, interleukin 1β, and Vcam1) in WI38 cells NC or Nrf2KO with or without rapamycin treatment (10 nm). *N* = 3–4 independent experiments. Statistical significance is indicated at *P < 0.05, **P < 0.01, and ^#^ *P* < 0.001.

To assess whether the mechanisms observed above using a stress‐induced premature senescence model is also observed in the more classical model of cellular senescence, we measured the role of Nrf2 in replicative senescence using WI38 human cells. As previously shown by Sell's group (Lerner *et al*., 2013), rapamycin delays replicative senescence and inhibit cell cycle arrest, but our data also indicate that the effect of rapamycin is dose‐dependent, which correlates with increased levels of Nrf2 and its downstream target gene NQO1, and a diminution in p16 levels (Fig. 4A,B). To determine the role of Nrf2 in this cellular model, we knocked down Nrf2 expression by Crispr–Cas9 technology. Our construct (NFE2L2 KO plasmid) encoded a GFP protein which facilitates the visualization/quantitation of the number of cells that have been targeted. Using this technique, we observed that between 83 and 89% of the cells were expressing GFP protein after 24 h of transfection (Fig. S6B, Supporting information). Higher transfection efficiency induced toxicity in WI38. As shown in Fig. 4C, knocked down Nrf2 accelerates replicative senescence, as compared to control cells transfected with noncoding gRNA (NC). Rapamycin treatment was able to delay the replicative senescence in both control and Nrf2KO WI38 cells (Fig. 4C). Because the number of cells decreases significantly after PD50, we decided to measure the senescent molecular markers, p16 and p21, by Western blot at 15 days (PD43). We observed a decreased in the levels of p16 and p21, only in WT cells, not in Nrf2 knockdown cells (Fig. 4D). To measure the effect on β‐gal, we used cells at 60 days (PD~46–48). In the WI38 model, Nrf2KO cells again showed higher levels of senescence as measured by β‐gal staining and cytokine levels, and rapamycin reduced the number of senescent cells in both WT and Nrf2KO WI38 cells, suggesting that the inhibition of β‐gal staining and secretory phenotype by rapamycin is independent of Nrf2 (Fig. 4E,F).

### Rapamycin decreases β‐gal staining and the SASP in Nrf2KO mice

**Figure 5:**
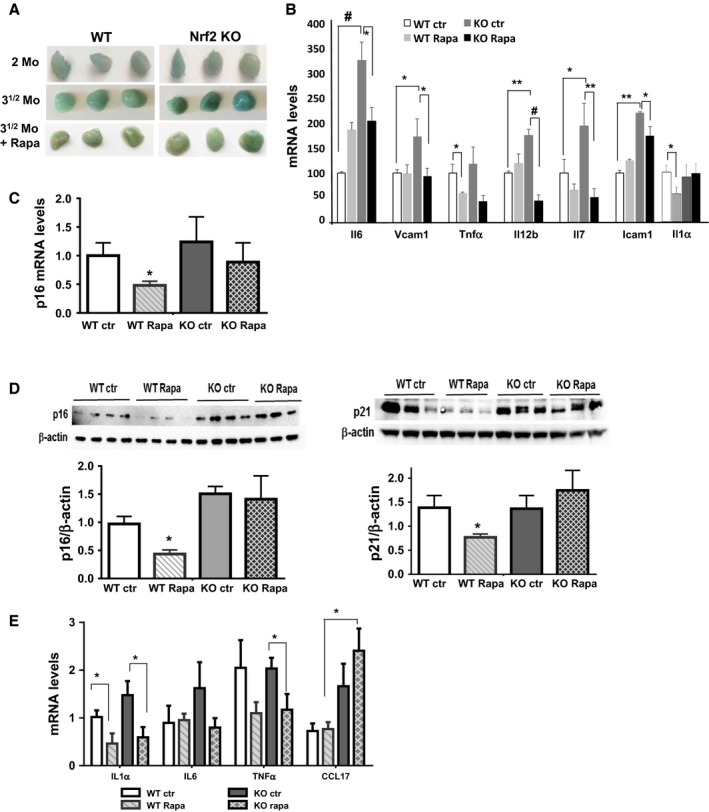
The effect of rapamycin on cellular senescence in fat and lung tissues from WT and Nrf2 null mice. (A) Representative β‐gal staining in fat tissues from WT and Nrf2KO mice at 2 months (2mo), or after 6 weeks (3^1/2^ mo) with or without rapamycin treatment. (B) Cytokines mRNA levels in fat tissues and (C) p16 mRNA levels were measured by qPCR analysis in fat tissues. (*N* = 6–8 for each group). (D) p16 and p21 protein levels in lung tissues from WT and Nrf2KO mice with or without rapamycin treatment. (E) Cytokines mRNA levels in lung tissues. *N* = 5–6 animals per group. Statistical significance is indicated at *P < 0.05; **P < 0.01, and ^#^ *P* < 0.001.

To determine whether the phenotype observed *in vitro* is also observed *in vivo*, we analyzed the same parameters in tissues from Nrf2KO mice treated with rapamycin. Rapamycin (4 mg kg^−1^ body weight) was administered to WT and Nrf2KO mice via i.p. injection every other day for 6 weeks (Fang *et al*., 2013), and several markers of cellular senescence were measured in fat and lung tissues. We found that in fat tissue from Nrf2KO mice at basal state showed higher levels of β‐gal staining and proinflammatory cytokines (measured by mRNA) than WT controls (Fig. 5A,B and Fig. S5, Supporting information). We observed that rapamycin treatment decreased β‐gal staining in fat tissue from both WT and Nrf2KO mice; however, rapamycin reduced the pro‐inflammatory cytokines mainly in the Nrf2KO mice, but much less in WT mice (Fig. 5A,B). In contrast, rapamycin did not reduce significantly the levels of p16 (measured by mRNA and immunohistochemistry) in fat tissue from Nrf2KO mice (Fig. 5C,D). Similar results were observed when we measured the protein levels of p16, p21, and mRNA levels of pro‐inflammatory cytokines in lung tissue (Fig. 5E), where rapamycin reduced p16 and p21 levels in WT but not in Nrf2KO mouse.

### Rapamycin inhibits the STAT pathway in a Nrf2‐independent manner

**Figure 6:**
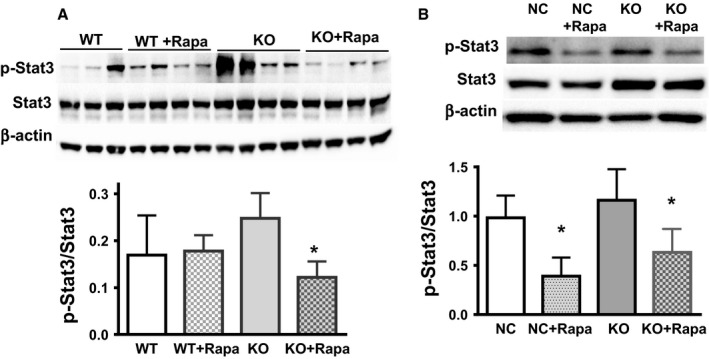
Rapamycin inhibits Stat3 activation in mouse lung tissue. STAT3 activation was measured by the ratio of P‐Stat3/Stat3 in (A) lung tissues from WT and Nrf2KO mice (*N* = 5–6 animals per group), and (B) WI38 (WT and Nrf2KO cells, *N* = 3). A representative Western blot is shown. Statistical significance is indicated at *P < 0.05.

Both cells and tissues from mice deficient in Nrf2 show increased levels of inflammation, characterized by high basal levels of cytokines. Evidence from the literature has shown that low grade, sterile inflammation is linked to activation of the JAK/Stat pathway. For example, inhibition of the JAK pathway by JAK inhibitors (Xu *et al*., 2015) rescues in part the SASP in senescent pre‐adipocytes *in vivo*. This effect was correlated with a significant reduction in the phosphorylation of Stat3. Based on this data, we decided to investigate if Stat3 is a potential pathway inhibited by rapamycin. Our data using lung tissue from Nrf2KO mice showed that tissue from Nrf2KO mice have increased basal levels of p‐Stat3, and rapamycin treatment reduced these levels (Fig. 6A). A similar effect was observed in WI38 cells deficient in Nrf2, which showed increased basal levels of the phosphorylated form of Stat3 (p‐Stat3), and rapamycin treatment was able to decrease these levels (Fig. 6B). However, we did not observe a significant change when we used MEF cells (Fig. S8B, Supporting information). These data suggest that JAK/Stat could at least partially explain the inhibitory effect of rapamycin on SASP induction in Nrf2KO mice.

## Discussion

Our study confirmed that rapamycin can prevent the induction of cell senescence by either genotoxic agents or by replicative exhaustion (Demidenko *et al*., 2009; Cao *et al*., 2011; Lerner *et al*., 2013). In addition, by identifying the role played by the Nrf2 pathway on these effects, we identify that the inhibition of cellular senescence by rapamycin can be molecularly dissected into effects on cell cycle arrest, and secretion of pro‐inflammatory molecules (SASP) and induction of senescence‐associated β‐galactosidase (β‐gal). We showed that rapamycin required Nrf2 to mediate the inhibition of cell cycle arrest, but did not require the Nrf2 to inhibit the SASP and β‐gal staining. Our data also showed that β‐gal staining is linked to the SASP rather than cell cycle arrest. These results were also corroborated *in vivo* using tissues from Nrf2KO mice, which displayed increased basal levels of cellular senescence. Again, rapamycin was able to reduce SASP and β‐gal staining, but not cell cycle arrest in this animal model.

The mechanisms by which cellular senescence is initiated have been widely studied; however, the mechanisms involved in the secretory activity of senescent cells are not well understood and has become a major focus in the field. Earlier studies using rapamycin as an inhibitor of cellular senescence focused on the effect on cell cycle arrest and β‐gal staining, where the decreased in 16/p21 were one of the parameters measured by several laboratories (Demidenko *et al*., 2009; Lerner *et al*., 2013, etc.). The interesting data shown by Campisi's and Gil's laboratories last year simply changed our vision about cellular senescence, as a disconnection was identified between these two arms, cell cycle arrest and β‐gal/SASP. Now, it does not necessarily mean that in some cells the two arms cannot run together; actually, this was the data that many studies have shown.

Nevertheless, these recent evidences have linked mTOR at the basis of the secretory activity of senescent cells. For example, studies by Herranz *et al*. (2015) and Laberge *et al*. (2015) have shown that inhibition of mTOR by rapamycin selectively inhibits the SASP and SA β‐gal staining of senescent cells without affecting their cell cycle arrest. These findings are novel and imply that in cell senescence, cell cycle arrest and the SASP are not coupled and therefore can be regulated independently. Mechanistically, Herranz *et al*. showed that mTOR activates the translation of MAPKAPK2 (MK2), a Ser/Thr‐kinase downstream of the MAP kinase p38, a well‐known inducer of cell senescence (Freund *et al*., 2010). It has been observed that MK2 stabilizes the mRNAs and upregulate the expression of many cytokines, and therefore, inhibition of MK2 by rapamycin results in a concomitant decrease in cytokine expression.

Similarly, the studies by Laberge *et al*. found that the translation of the mRNA for IL1α is highly dependent on mTOR activity. When IL1α is expressed and secreted, it triggers the transcription of the master inflammatory transcription factor NF(B in both paracrine and autocrine fashions, which ultimately will activate multiple cytokine‐encoding genes. These data could explain how the inhibition of mTOR by rapamycin decreases the expression of IL1α as well as the expression of several other cytokines and is consistent with previous observations *in vivo*, where rapamycin impairs the SASP, decreases the recruitment of inflammatory cells, prevents the age‐related loss of tissue function, delays/ameliorates several age‐related diseases, and extends health/lifespan in mice.

Recently, Correia‐Melo *et al*. (2016) reported that DNA damage response (DDR) [induced by stress (i.e., ROS) or cell replication] activates mitochondrial biogenesis which will mediate a cell cycle arrest. Interestingly, they showed that the reduction in mitochondrial content by mTORC1 inhibition prevents cellular senescence. This can explain why Nrf2KO mice (that have high levels of oxidative stress and abnormal mitochondria) showed enhanced p16/p21 pathway and also suggest a new mechanism by which rapamycin may suppress cellular senescence and uncouple it from cell division.

Therefore, cell senescence is a very complex process that involves at least two arms, cell cycle arrest and the secretory phenotype, and these can be regulated independently at many levels (from transcriptional to paracrine/autocrine signaling pathways). Several recent observations also demonstrated that SA β‐gal is more closely linked to the SASP than to cell cycle arrest. Although it is not known how the inhibition of mTOR ameliorates the SA β‐gal, some evidence suggests that SA β‐gal staining is associated with an increase in the mTOR pathway and activation of lysosome activity, but it is not necessary linked to an activation of autophagy, which seems to be down regulated in cell senescence (Pena‐Llopis & Brugarolas, 2011).

The actual mechanism by which rapamycin increases Nrf2 levels is not completely elucidated; however, this seems to occur at the level of protein stabilization rather than increasing Nrf2 gene expression because rapamycin did not increase Nrf2 mRNA levels, but decreased Keap1, a protein that triggers Nrf2 degradation (Kobayashi *et al*., 2004). Based on previous studies in the aging field, it is believed that Nrf2 is repressed by mTOR pathway (downstream target); therefore, an inhibition of mTOR by rapamycin would increase the activity of Nrf2 (Johnson *et al*., 2013). However, recently an interesting study has shown that increased levels of Nrf2 can directly activate transcription of the mTOR gene and activate the PI3K signaling pathway. However, this happens only in cells that have a normal physiological PI3K activity (Bendavit *et al*., 2016). All these studies confirm that the mTOR pathway is involved in a very complex network, which will change depending on the metabolic state of the cells.

Our data also confirm that rapamycin inhibits but does not reverse cellular senescence, because the levels of β‐gal staining in fat tissue are similar between animals treated with rapamycin and their controls. This explains why pre‐incubation with rapamycin for 24 h before SIPS (H_2_O_2_) had better results in the inhibition of cellular senescence (Fig. S1, Supporting information) than co‐incubation with SIPS.

An early study using Nrf2KO mice showed that these mice suffer from several aspects of the aging phenotype, such as alopecia, kyphosis, and a short lifespan, suggesting that they suffer from a premature aging phenotype. Although Nrf2KO mice at young age indeed showed an impaired response to oxidative stress and develop evidence of several chronic diseases such as emphysema, cancer, gastritis, retinopathy, and increased inflammation (Ishii *et al*., 2005; Khor *et al*., 2008), these animals are viable and developed normally. However, a report from de Cabo's laboratory showed that the Nrf2 pathway mediates cancer protection but not the pro‐longevity induced by caloric restriction and that the Nrf2KO have a normal lifespan relative to their wild‐type counterparts (Pearson *et al*., 2008).

Our data show that tissue and plasma from Nrf2KO mice present higher levels of inflammation at 4 month of age (measured by the cytokine levels), corroborating previous reports (Ishii *et al*., 2005; Khor *et al*., 2008). This inflammatory status was attenuated by rapamycin treatment, and the inhibition of the inflammatory phenotype by rapamycin was correlated with an inhibition of the Stat3 pathway in both cell and mouse models (Xu *et al*., 2015). By measuring the levels of p65/p50 in the nucleus vs. cytosol (Fig. S6, Supporting information), we also observed that rapamycin inhibited the NF‐κB pathway only in MEF from WT but not from Nrf2KO MEF. This is consistent with the notion that Nrf2 regulates NF‐κB activation; however, we did not see significant changes in the NF‐κB pathway in tissues from Nrf2KO mice (measured by p65/p50 translocation). We did observe a significant decrease in pro‐inflammatory cytokine levels, suggesting that another pro‐inflammatory pathway may be inhibited by rapamycin even in the absence of Nrf2.

Based on recently published data, we believe that while cell cycle arrest is mediated by Nrf2 (and inhibited by rapamycin), the inhibition of SASP is dependent on mTOR activation in a manner that is independent of Nrf2. Inhibition of the SASP probably requires activation of MAPKAPK2, which would explain the decrease in pro‐inflammatory cytokines. In fact, inhibition of MAPKAPK2 by rapamycin has been shown to decrease the levels of pro‐inflammatory cytokines including IL1α, and this effect is highly dependent on mTOR activity (Laberge *et al*., 2015; Herranz *et al*., 2015). However, we must to understand that the SASP can vary significantly in terms of the quantity and quality of secreted proteins and other factors, depending on the insult used to trigger cell senescence (Maciel‐Baron *et al*., 2016; Wiley *et al*., 2016); therefore, the composition of the SASP may be vary depending on the tissue and the insult.

In summary, our data show that inhibition of the SASP by rapamycin is independent of the Nrf2 pathway and does not require a cell cycle arrest (see our current model in Fig. S7, Supporting information). This is important because it is believed that the secretory phenotype of senescent cells is a major starting point in the chronic sterile inflammation that correlates with several age‐related diseases, as well as the anti‐aging effects observed upon removal of senescent cells (Baker *et al*., 2016). However, because it has also been shown that removal of senescent cells has some negative effects, such as in wound healing (Demaria *et al*., 2014), it seems that suppression of the SASP by rapamycin, but without the actual removal of senescent cells may have broader beneficial effects as a therapy against age‐related diseases.

## Experimental procedures

### Cell culture and treatment

MEF cells were maintained in IMDM medium (Invitrogen, Grand Island, NY, USA), and primary skin fibroblasts were obtained from C57/B6 mouse and were maintained in DMEM with low glucose medium (Invitrogen). Human WI38 cells were maintained in EMEM medium (Corning Cellgro, Manassa, VA, USA). All cultures were supplemented with 10% FBS, 100 units mL^−1^ penicillin, and 100 μg mL^−1^ streptomycin at 37 °C in 5% CO_2_.

#### Stress‐induced premature senescence (SIPS)

MEF and skin fibroblast cells were pretreated with rapamycin (25 nm for MEF, 250 nm for skin fibroblasts) or DMSO vehicle as control for 24 h. Cells were incubated with H_2_O_2_ for 2 h (1 μm for MEF, 150 nm for skin fibroblasts). After washing with PBS, cells were maintained in media with rapamycin or DMSO for 1, 2, 4, or 6 days and harvested for mRNA and protein expression analyses.

#### Replicative senescence

WI38 cells were incubated with rapamycin (2 or 10 nm), or DMSO vehicle as control. Cumulative population doubling (PD) level was calculated according to the following equation: PD = X + 3.322(LogY‐LogI), X = initial population doubling level, I = initial cell number, Y = the cell number at the end of the growth period (Lerner *et al*., 2013).

### SA‐β‐galactosidase (β‐gal) staining

Cell SA‐β‐gal (β‐gal) staining was performed with Senescence β‐Galactosidase Staining Kit (Cell Signaling,Danvers, MA, USA, #9860) following manufacturer's instruction. The blue‐stained cells were counted in 10 fields under the microscope with 200× magnification and expressed as a percentage of positive cells. To avoid staining due to cell confluence rather than to proliferative senescence (Severino *et al*., 2000), the assay was performed in subconfluent cultures displaying comparable cell density. Fat tissue β‐gal staining was performed immediately after mice were euthanized; tissues were cut into small pieces and stained as described by manufacturer's instructions.

### Overexpression with Nrf2

Cells (1.5 × 10^5^) were transfected with 1 μg Nrf2 cDNA (Addgene, Cambridge, MA, USA) or empty plasmid DNA. At day 2, cells were pretreated with rapamycin 25 nm or DMSO. At day 3, cells were exposure to H_2_O_2_ for 2 h. After washing, cells were incubated with rapamycin 25 nm. At days 4 and 7, transfection was repeated with 1 μg DNA. At day 9, cells were stained with β‐gal or harvested for immunoblotting analysis.

### Knockout of Nrf2 using Crispr/Cas9 technique

The CRISPR/CAS9 system was utilized to knock out the NFE2L2 gene. 1.5 × 10^5^ WI38 cells were seeded in 6‐well plate overnight (day 0) and transfected with 1.0 μg of NFE2L2 KO plasmid (DNA 2.0, Menlo, CA, USA) that encoded GFP, Nickase Cas9 protein, and two guide RNAs that targeted DNA mutations specifically to a protein coding exon shared by the NFE2L2 isoforms (exon 2). Alternatively, negative control cells were transfected with the same plasmid except the guide RNAs targeted DNA mutations to a region of DNA on chromosome 13 where no genes are present; 24 h following transfection cells were verified to be GFP positive (80% transfection efficiency) and treated with rapamycin 10 nm and maintained with rapamycin until they were harvested for mRNA, protein expression analysis, and β‐gal staining.

### Cytokine antibody array

Cell‐conditioned media from wild‐type and Nrf2 null MEF cells treated 6 days with rapamycin or DMSO were collected after 2 days in serum free media. Proinflammatory factor levels were measured using mouse cytokine antibody array (RayBio^®^ C2000, Norcross, CA, USA) according to the manufacturer's instructions. The intensities of signals were quantified, normalized to positive control, and calculated as log_2_‐fold changes relative to untreated wild‐type MEF (baseline).

### Animals

This study received prior approval from Oregon State University Institutional Animal Care and Use Committee. Two‐month‐old WT and Nrf2 knockout mice (ICR background, obtained from Dr. Masayuki, Yamamoto Kohoku University of Japan) were divided into four groups including wild‐type (WT) and Nrf2 KO mice with or without rapamycin treatment (each group contained four male and four female mice). The mice received i.p. with rapamycin at a dose of 4 mg kg^−1^ body weight (dissolved in ethanol then diluted with vehicle containing 5% Tween‐80 and 5% PEG400) or same volume ethanol diluted with vehicle as controls. The dosing was given at every other day for 6 weeks (Fang *et al*., 2013). The mice were euthanized at 48 h after last dose of rapamycin and a thorough necropsy was performed. Tissues were snap frozen in liquid nitrogen, then stored at −80 °C for subsequent molecular work.

### Quantitative real time PCR

The mRNA was extracted using the RNeasy kit (Qiagen, Valencia, CA, USA). RNA was reverse‐transcribed to cDNA using SuperScript^®^ III First‐Strand Synthesis SuperMix following manufacturer's instruction (Invitrogen). Target mRNA levels were measured by qPCR and normalized to actin beta (actb). The amount of specific mRNA was quantified by determining the point at which the fluorescence accumulation entered the exponential phase (*C*_t_), and the *C*_t_ ratio of the target gene to actb was calculated for each sample. The information for qPCR primers is listed in Appendix S1.

### Western blotting

20 μg of protein was subjected by SDS‐PAGE and processed by immunoblotting. The proteins were visualized by Western Lightning™ chemiluminescence Reagent Plus (PerkinElmer Life Sciences, Boston MA, USA) and quantified using Bio‐Rad Image System (Hercules, CA, USA). The information for antibodies is listed in Appendix S1.

### Statistics

All *in vitro* experiments were repeated at least three times. Data were analyzed by one‐way or two‐way ANOVA with post hoc analysis by Holm‐Sidak or by Student's *t*‐test where appropriate. Data are presented as mean ± SEM, and statistical significance is indicated at *P*‐value less than *P < 0.05; **P < 0.01; and #*P* < 0.01.

## Funding

Financial support was provided by American Federation for Aging Research (AFAR) (V.P) and funds from The Linus Pauling Institute, and Biochemistry and Biophysics Department, Oregon State University.

## Conflict of interest

The authors have no financial conflict of interest to declare.

## Author contributions

R.W. and V.I.P. designed the research; R.W., Z.Y., B.S., J.S., I.D., S.Z., K.C., L.B., and L.M.B. performed research; R.W., C.V.L, B.S., E.H., and V.I.P. analyzed data; R.W., and V.I.P. wrote the manuscript.

## Supporting information


**Fig. S1** Pre‐incubation with rapamycin have better effects on Nrf2 expression and inhibition of cell senescence.
**Fig. S2** Overexpression of Nrf2 in MEFs recues the NQO1 expression (A).
**Fig. S3** Measurement of p16 in skin fibroblasts and MEFs using different antibodies.
**Fig. S4** Rapamycin‐induced autophagy in MEF cells is dependent on Nrf2.
**Fig. S5** Cell proliferation in MEF cells: wild type (WT), Nrf2KO (KO), Nrf2 KO cells transfected with empty vector (KO+vec) or Nrf2 cDNA (KO+Nrf2) in control cells and in response to SIPS or SIPS + Rapa, was measured by BrdU ELISA assay (colorimetric) using 2 hrs of pulse length (A) and by BrdU immunocytochemistry staining (B).
**Fig. S6** b‐gal staining was performed in Nrf2KO MEF control cells and in response to SIPS or SIPS+ Rapa after 6 days of post‐treatment.
**Fig. S7** Images showing the b‐gal staining in fat tissue from WT and Nrf2KO mice.
**Fig. S8** Inhibition of mTOR pathway by different doses of rapamycin in WT and Nrf2KO MEF control cells, in response to SIPS, or SIPS+Rapa after 6 days of rapamycin posttreatment.
**Fig. S9** Current model to explain the mechanisms by which rapamycin inhibit cell senescence in a Nrf2 dependent and independent manner.

**Appendix S1** Experimental procedure.
