# Supplementary material for: Rapamycin inhibits the secretory phenotype of senescent cells by a Nrf2‐independent mechanism
Source: Aging Cell. 2017 Mar 31;16(3):564–74. doi: 10.1111/acel.12587 (PMC5418203; doi:10.1111/acel.12587)
Supplement: Supplementary file 1 — Fig. S1 Pre‐incubation with rapamycin have better effects on Nrf2 expression and inhibition of cell senescence. Fig. S2 Overexpression of Nrf2 in MEFs recues the NQO1 expression (A). Fig. S3 Measurement of p16 in skin fibroblasts and MEFs using different antibodies. Fig. S4 Rapamycin‐induced autophagy in MEF cells is dependent on Nrf2. Fig. S5 Cell proliferation in MEF cells: wild type (WT), Nrf2KO (KO), Nrf2 KO cells transfected with empty vector (KO+vec) or Nrf2 cDNA (KO+Nrf2) in control cells and in response to SIPS or SIPS + Rapa, was measured by BrdU ELISA assay (colorimetric) using 2 hrs of pulse length (A) and by BrdU immunocytochemistry staining (B). Fig. S6 b‐gal staining was performed in Nrf2KO MEF control cells and in response to SIPS or SIPS+ Rapa after 6 days of post‐treatment. Fig. S7 Images showing the b‐gal staining in fat tissue from WT and Nrf2KO mice. Fig. S8 Inhibition of mTOR pathway by different doses of rapamycin in WT and Nrf2KO MEF control cells, in response to SIPS, or SIPS+Rapa after 6 days of rapamycin posttreatment. Fig. S9 Current model to explain the mechanisms by which rapamycin inhibit cell senescence in a Nrf2 dependent and independent manner. [file ACEL-16-564-s001.pptx]

## Slide 1
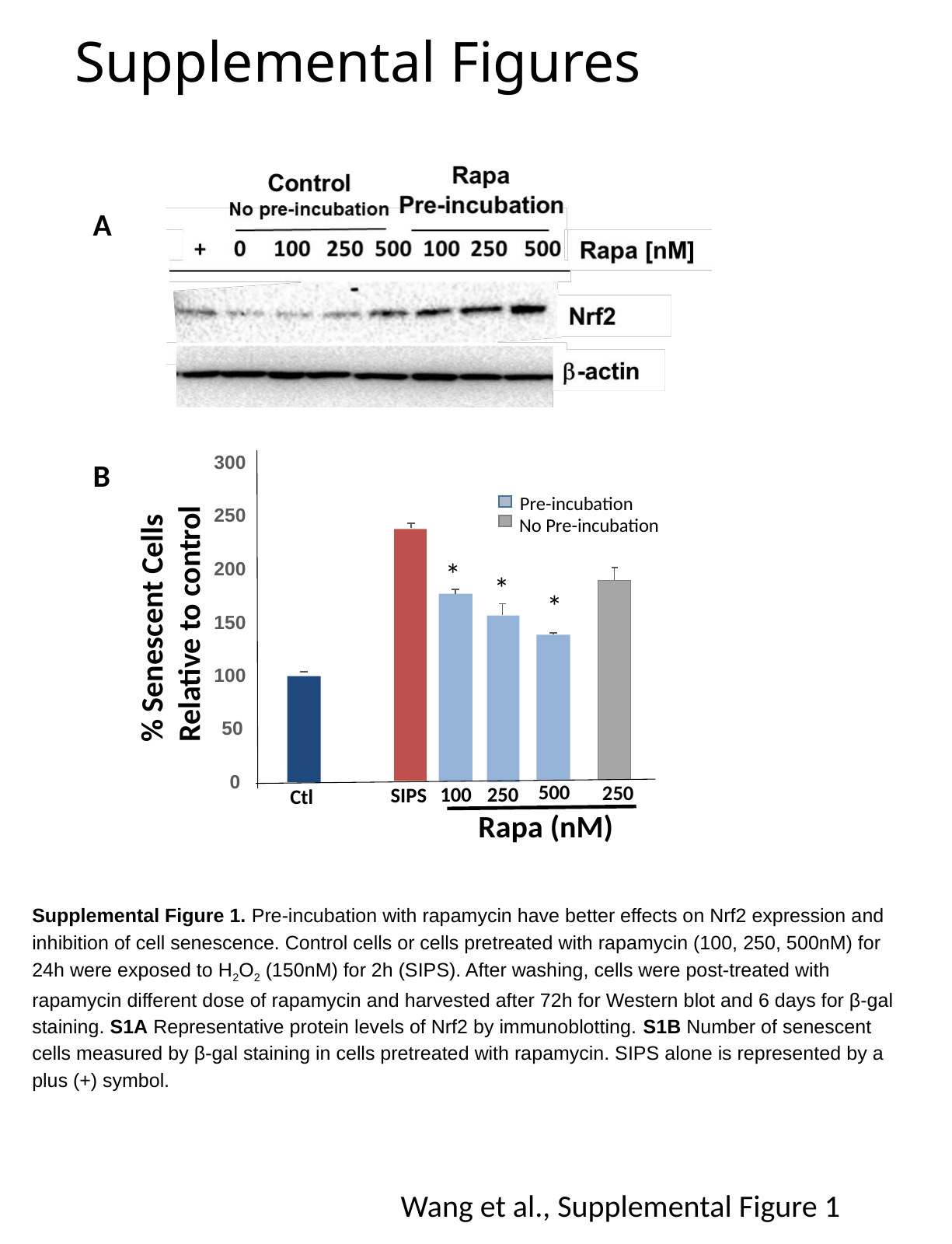

# Supplemental Figures
A
300
Pre-incubation
No Pre-incubation
250
*
200
*
*
150
100
50
0
500
250
100
250
SIPS
Ctl
Rapa (nM)
B
% Senescent Cells
Relative to control
Supplemental Figure 1. Pre-incubation with rapamycin have better effects on Nrf2 expression and inhibition of cell senescence. Control cells or cells pretreated with rapamycin (100, 250, 500nM) for 24h were exposed to H2O2 (150nM) for 2h (SIPS). After washing, cells were post-treated with rapamycin different dose of rapamycin and harvested after 72h for Western blot and 6 days for β-gal staining. S1A Representative protein levels of Nrf2 by immunoblotting. S1B Number of senescent cells measured by β-gal staining in cells pretreated with rapamycin. SIPS alone is represented by a plus (+) symbol.
Wang et al., Supplemental Figure 1

## Slide 2
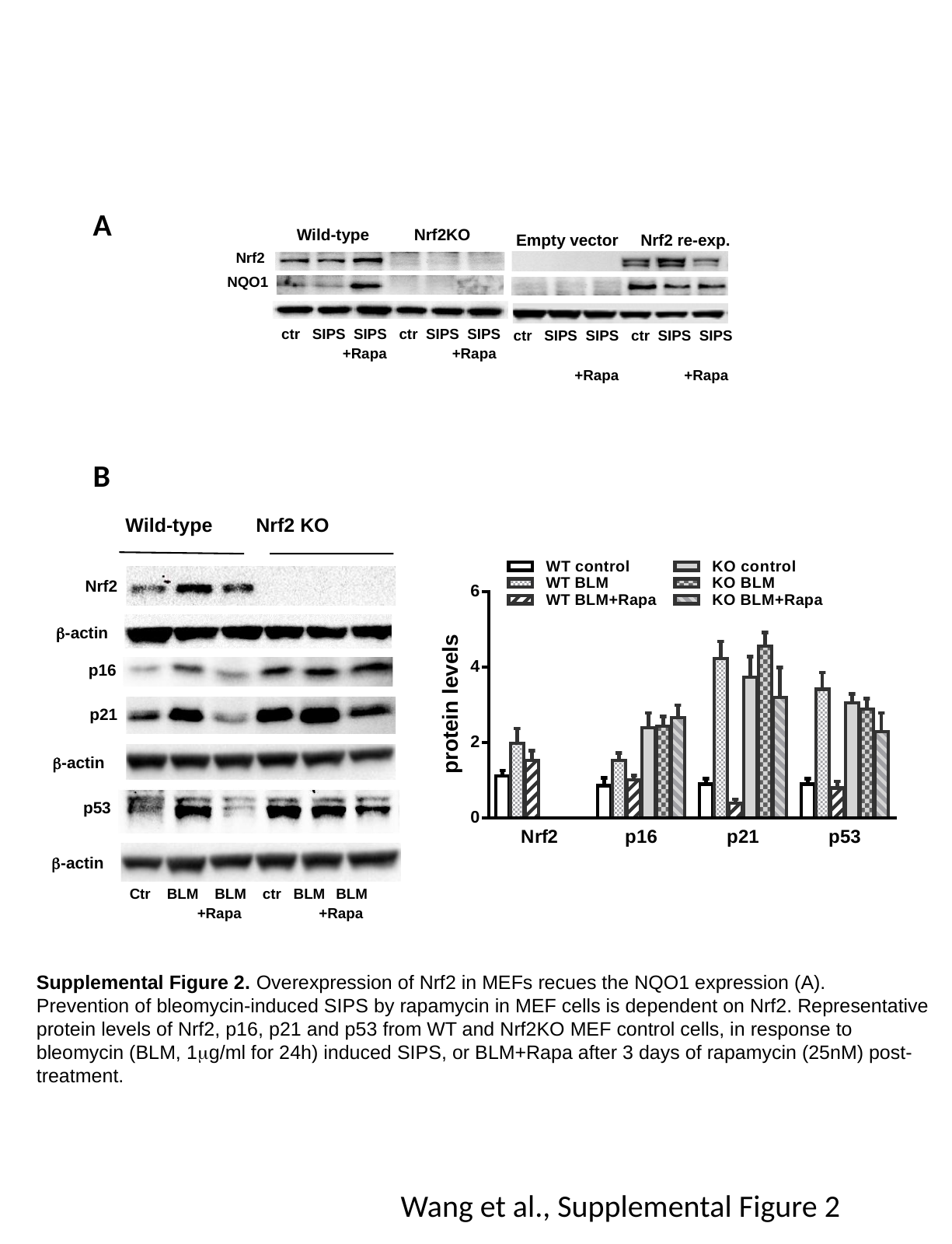

A
Wild-type Nrf2KO
Empty vector Nrf2 re-exp.
Nrf2
NQO1
 ctr SIPS SIPS ctr SIPS SIPS
 +Rapa +Rapa
 ctr SIPS SIPS ctr SIPS SIPS
 +Rapa +Rapa
B
Wild-type Nrf2 KO
Nrf2
b-actin
p16
p21
b-actin
p53
b-actin
Ctr BLM BLM ctr BLM BLM
 +Rapa +Rapa
Supplemental Figure 2. Overexpression of Nrf2 in MEFs recues the NQO1 expression (A). Prevention of bleomycin-induced SIPS by rapamycin in MEF cells is dependent on Nrf2. Representative protein levels of Nrf2, p16, p21 and p53 from WT and Nrf2KO MEF control cells, in response to bleomycin (BLM, 1mg/ml for 24h) induced SIPS, or BLM+Rapa after 3 days of rapamycin (25nM) post-treatment.
Wang et al., Supplemental Figure 2

## Slide 3
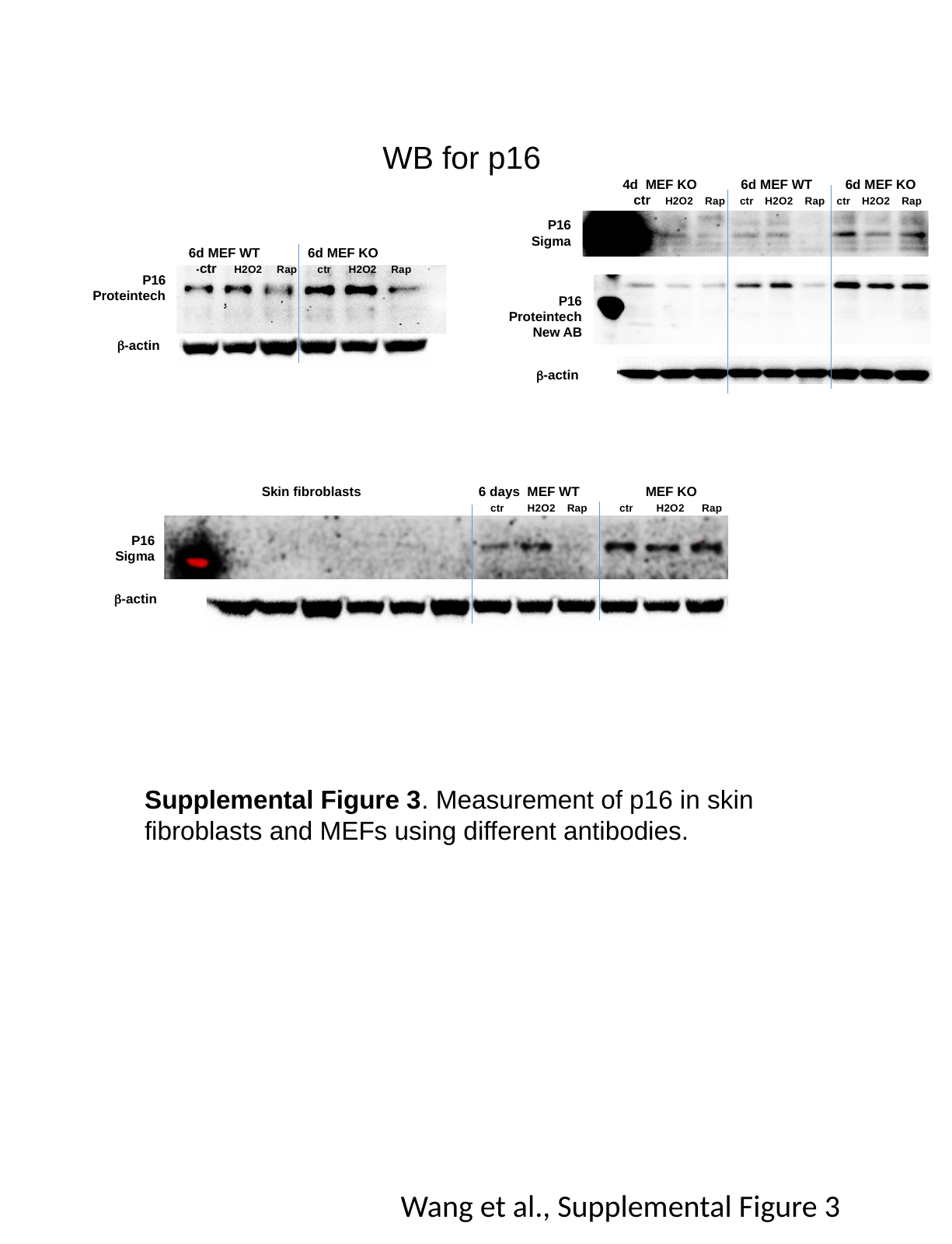

WB for p16
4d MEF KO 6d MEF WT 6d MEF KO
 ctr H2O2 Rap ctr H2O2 Rap ctr H2O2 Rap
P16
Sigma
6d MEF WT 6d MEF KO
 ctr H2O2 Rap ctr H2O2 Rap
P16
Proteintech
P16
Proteintech
New AB
b-actin
b-actin
Skin fibroblasts 6 days MEF WT MEF KO
 ctr H2O2 Rap ctr H2O2 Rap
P16
Sigma
b-actin
Supplemental Figure 3. Measurement of p16 in skin fibroblasts and MEFs using different antibodies.
Wang et al., Supplemental Figure 3

## Slide 4
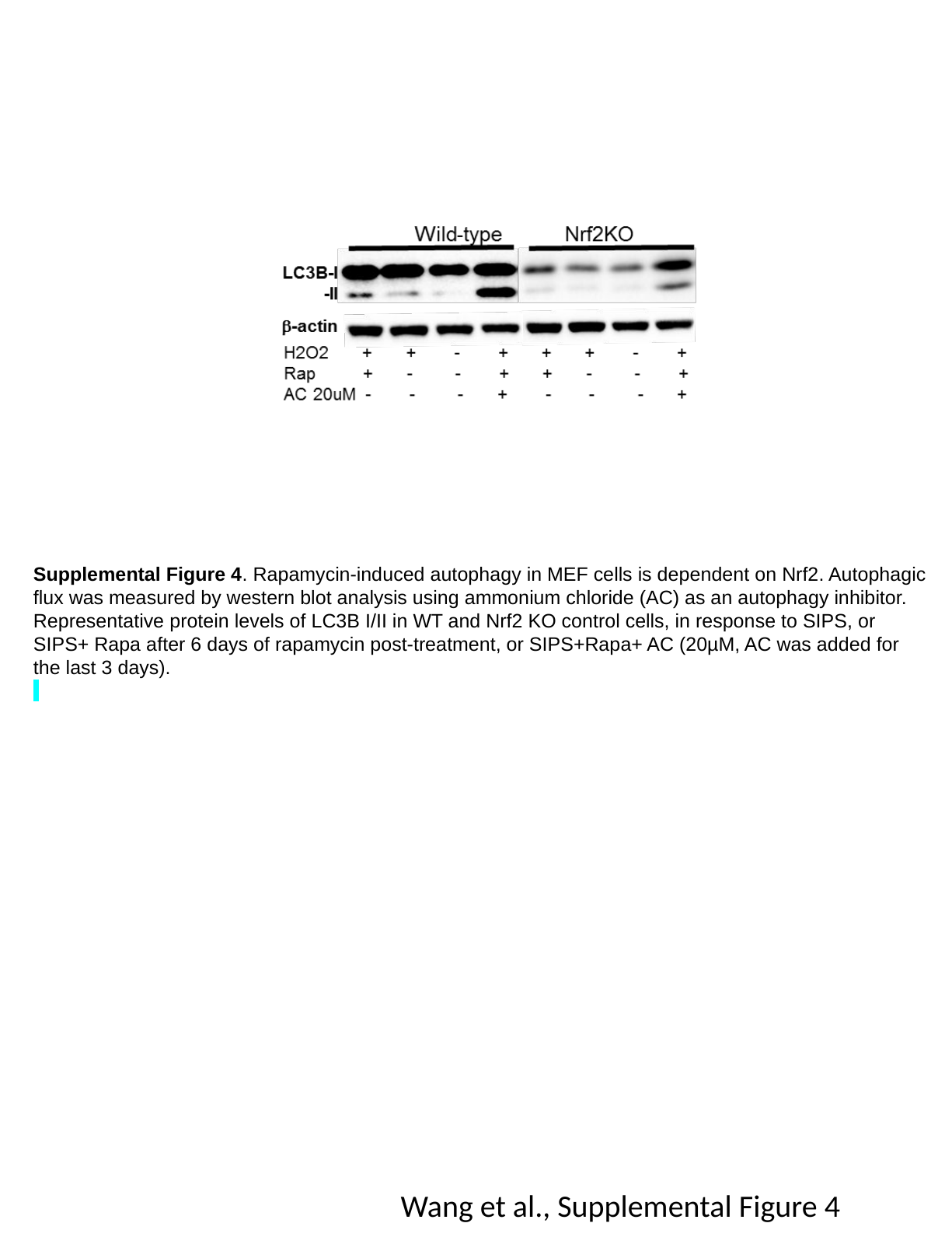

Supplemental Figure 4. Rapamycin-induced autophagy in MEF cells is dependent on Nrf2. Autophagic flux was measured by western blot analysis using ammonium chloride (AC) as an autophagy inhibitor. Representative protein levels of LC3B I/II in WT and Nrf2 KO control cells, in response to SIPS, or SIPS+ Rapa after 6 days of rapamycin post-treatment, or SIPS+Rapa+ AC (20µM, AC was added for the last 3 days).
Wang et al., Supplemental Figure 4

## Slide 5
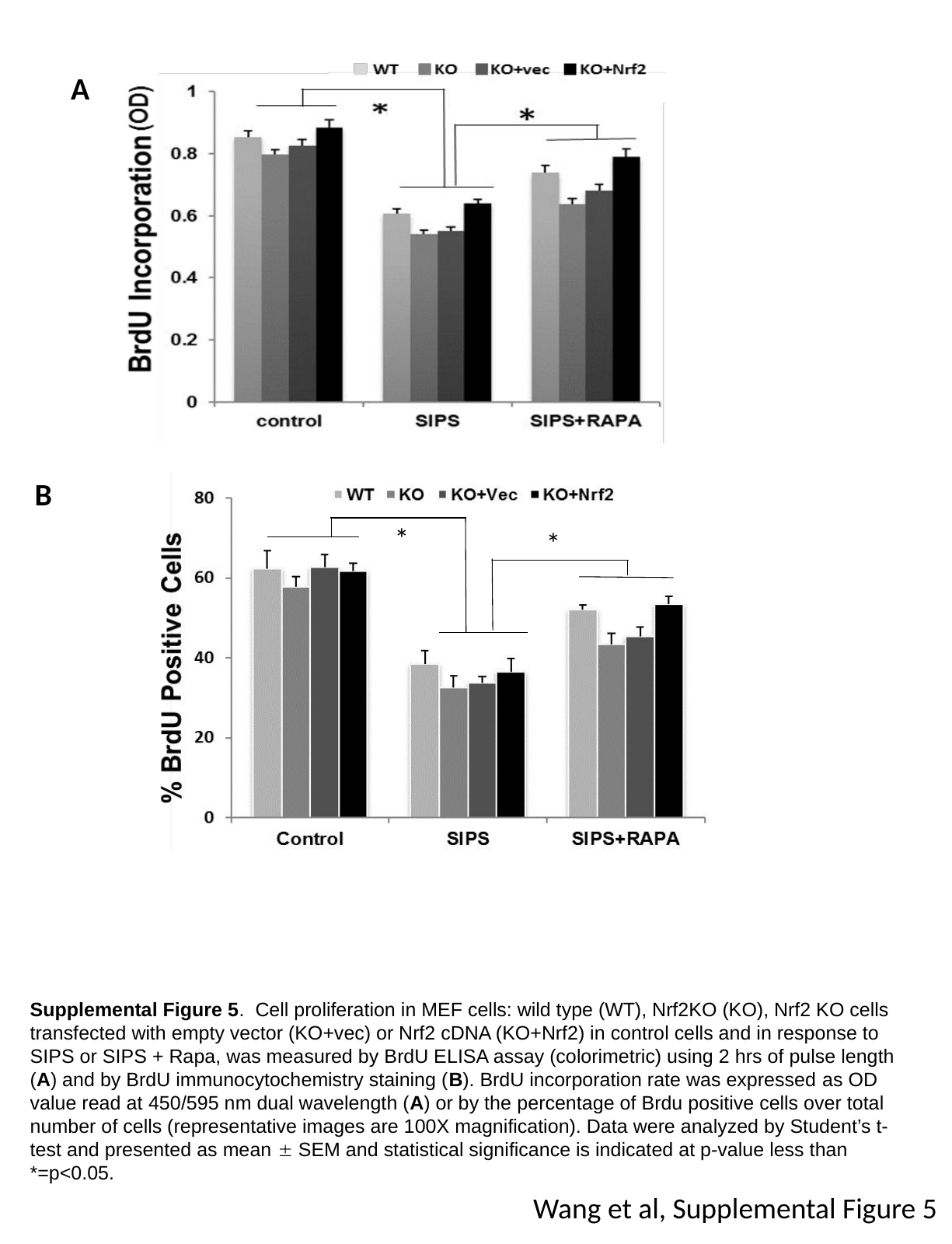

A
B
*
*
Supplemental Figure 5. Cell proliferation in MEF cells: wild type (WT), Nrf2KO (KO), Nrf2 KO cells transfected with empty vector (KO+vec) or Nrf2 cDNA (KO+Nrf2) in control cells and in response to SIPS or SIPS + Rapa, was measured by BrdU ELISA assay (colorimetric) using 2 hrs of pulse length (A) and by BrdU immunocytochemistry staining (B). BrdU incorporation rate was expressed as OD value read at 450/595 nm dual wavelength (A) or by the percentage of Brdu positive cells over total number of cells (representative images are 100X magnification). Data were analyzed by Student’s t-test and presented as mean ± SEM and statistical significance is indicated at p-value less than *=p<0.05.
Wang et al, Supplemental Figure 5

## Slide 6
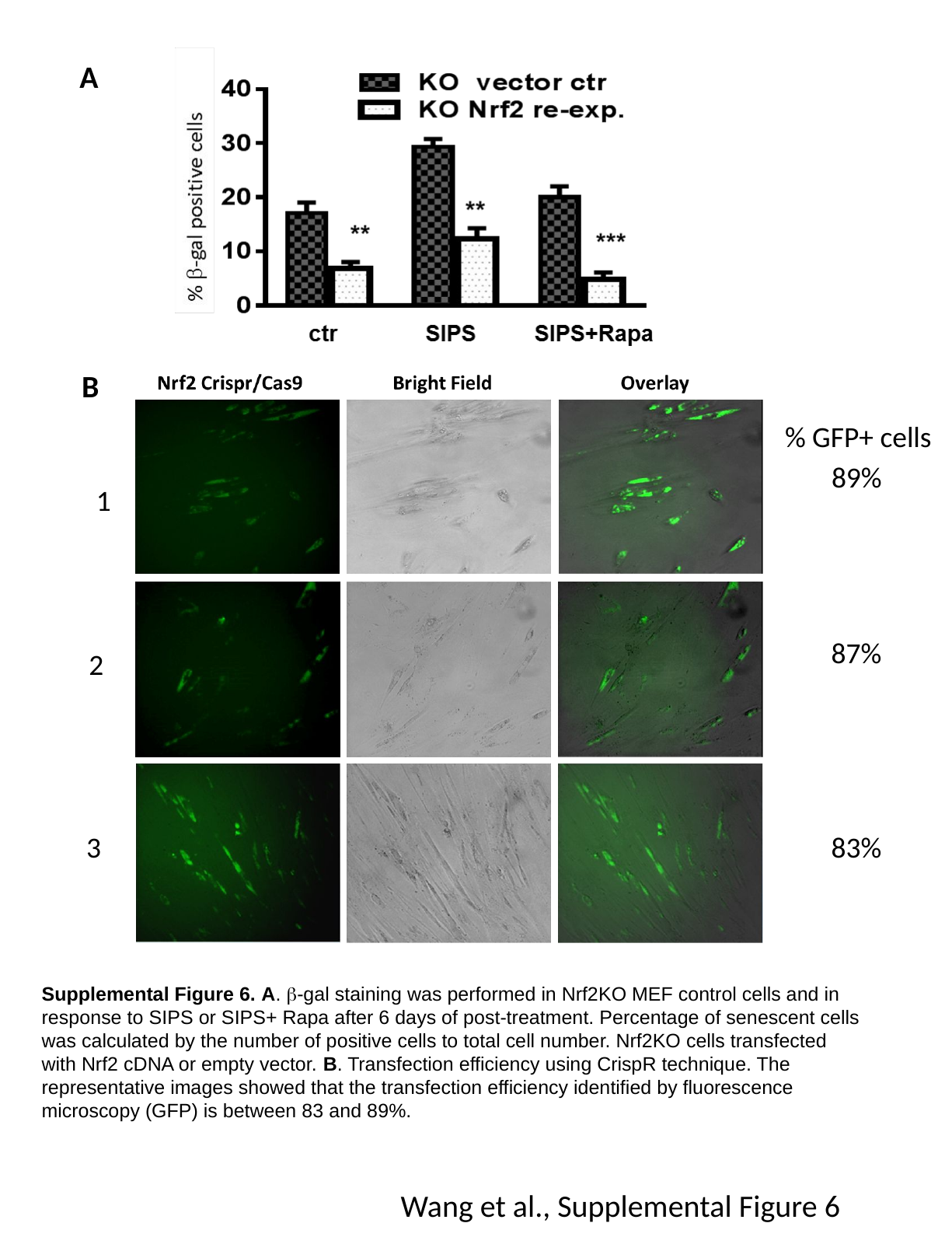

A
B
% GFP+ cells
89%
1
87%
2
3
83%
Supplemental Figure 6. A. b-gal staining was performed in Nrf2KO MEF control cells and in response to SIPS or SIPS+ Rapa after 6 days of post-treatment. Percentage of senescent cells was calculated by the number of positive cells to total cell number. Nrf2KO cells transfected with Nrf2 cDNA or empty vector. B. Transfection efficiency using CrispR technique. The representative images showed that the transfection efficiency identified by fluorescence microscopy (GFP) is between 83 and 89%.
Wang et al., Supplemental Figure 6

## Slide 7
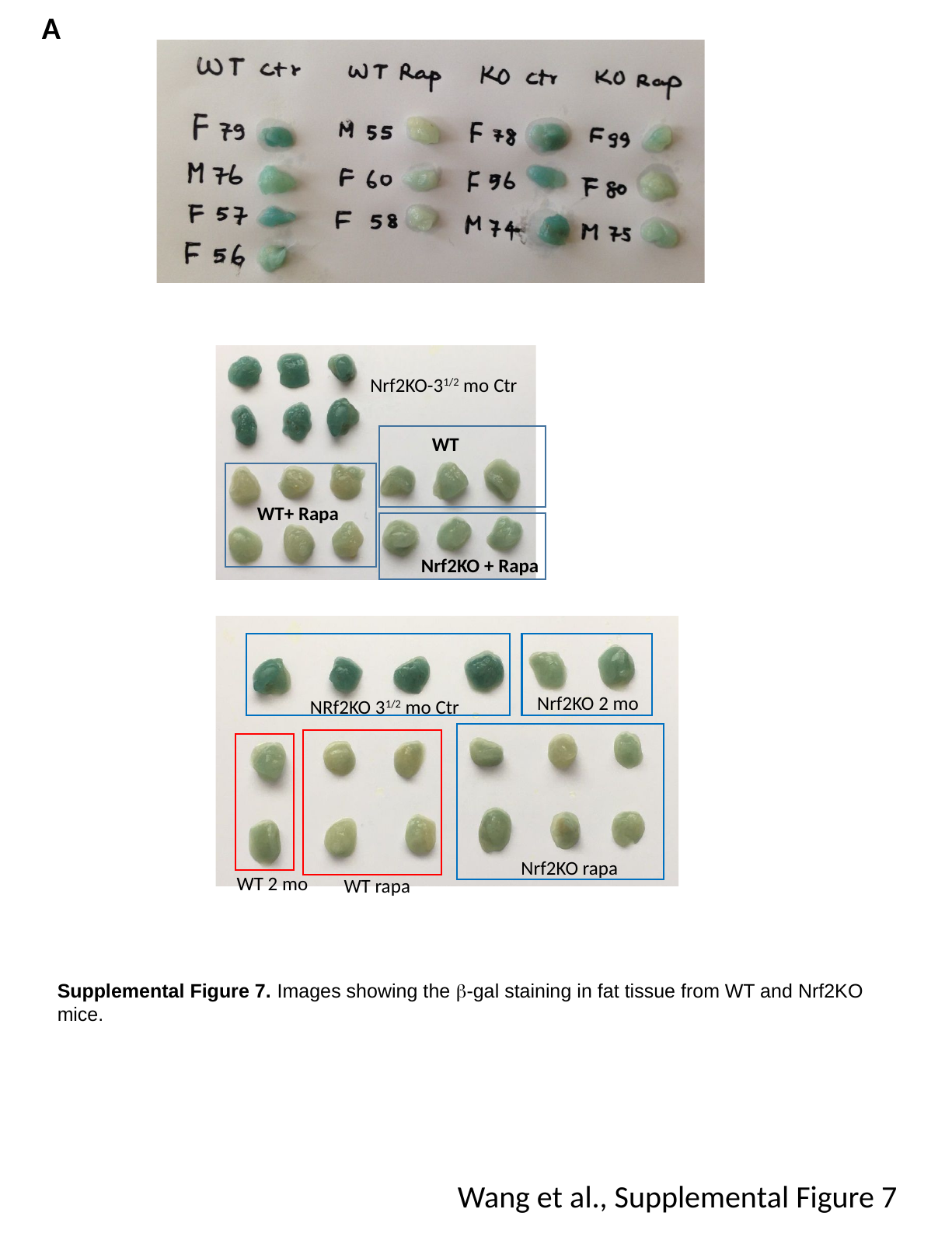

A
Nrf2KO-31/2 mo Ctr
WT
WT+ Rapa
Nrf2KO + Rapa
Nrf2KO 2 mo
NRf2KO 31/2 mo Ctr
Nrf2KO rapa
WT 2 mo
WT rapa
Supplemental Figure 7. Images showing the b-gal staining in fat tissue from WT and Nrf2KO mice.
Wang et al., Supplemental Figure 7

## Slide 8
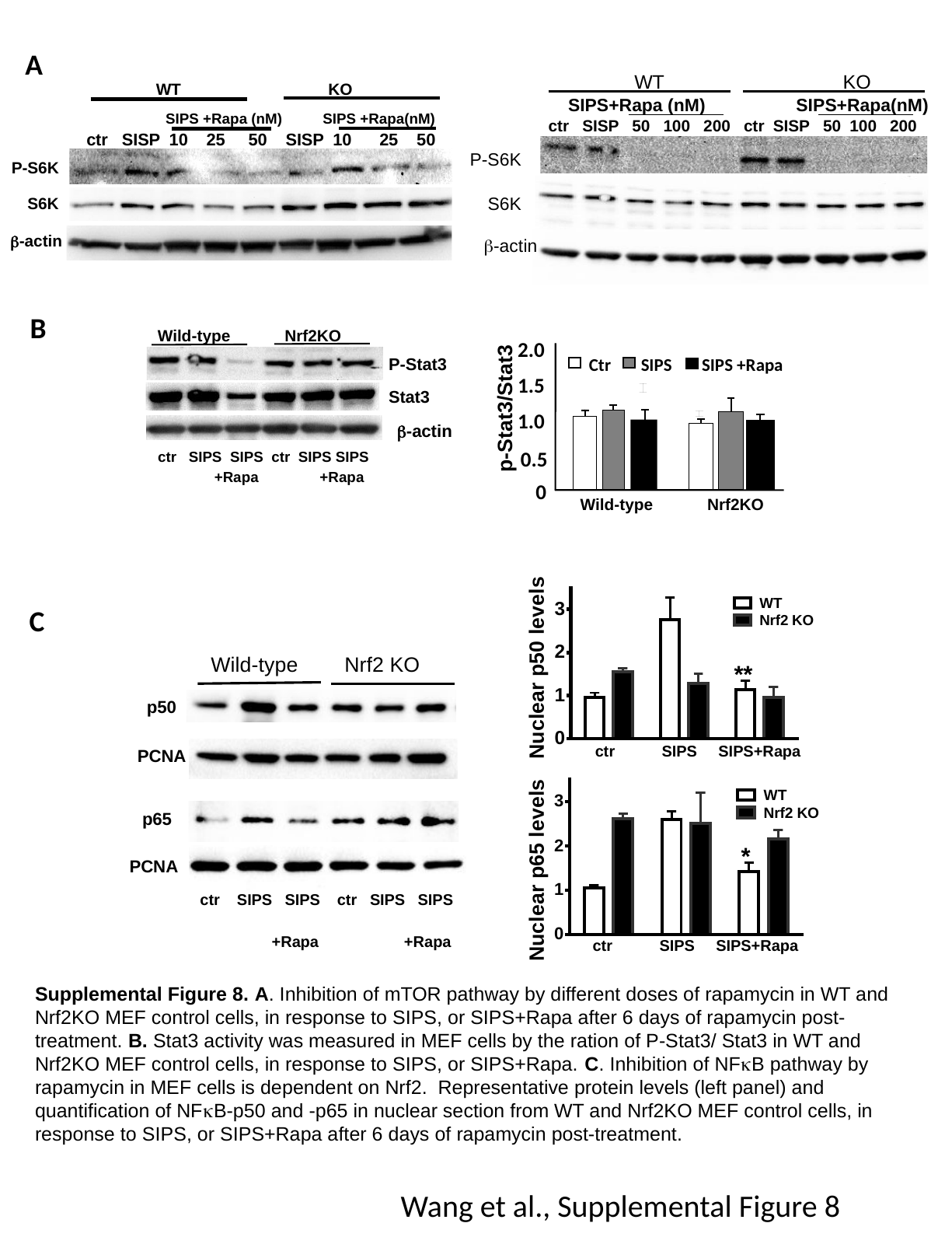

A
WT KO
SIPS+Rapa (nM) SIPS+Rapa(nM)
ctr SISP 50 100 200 ctr SISP 50 100 200
P-S6K
S6K
b-actin
 WT KO
SIPS +Rapa (nM) SIPS +Rapa(nM)
ctr SISP 10 25 50 SISP 10 25 50
P-S6K
S6K
b-actin
B
Wild-type Nrf2KO
P-Stat3
Stat3
 ctr SIPS SIPS ctr SIPS SIPS
 +Rapa +Rapa
2.0
1.5
1.0
0.5
0
Ctr
SIPS
SIPS +Rapa
 p-Stat3/Stat3
Wild-type Nrf2KO
b-actin
C
Nuclear p50 levels
Wild-type Nrf2 KO
**
p50
 ctr SIPS SIPS+Rapa
PCNA
p65
*
Nuclear p65 levels
PCNA
 ctr SIPS SIPS ctr SIPS SIPS
 +Rapa +Rapa
 ctr SIPS SIPS+Rapa
Supplemental Figure 8. A. Inhibition of mTOR pathway by different doses of rapamycin in WT and Nrf2KO MEF control cells, in response to SIPS, or SIPS+Rapa after 6 days of rapamycin post-treatment. B. Stat3 activity was measured in MEF cells by the ration of P-Stat3/ Stat3 in WT and Nrf2KO MEF control cells, in response to SIPS, or SIPS+Rapa. C. Inhibition of NFkB pathway by rapamycin in MEF cells is dependent on Nrf2. Representative protein levels (left panel) and quantification of NFkB-p50 and -p65 in nuclear section from WT and Nrf2KO MEF control cells, in response to SIPS, or SIPS+Rapa after 6 days of rapamycin post-treatment.
Wang et al., Supplemental Figure 8

## Slide 9
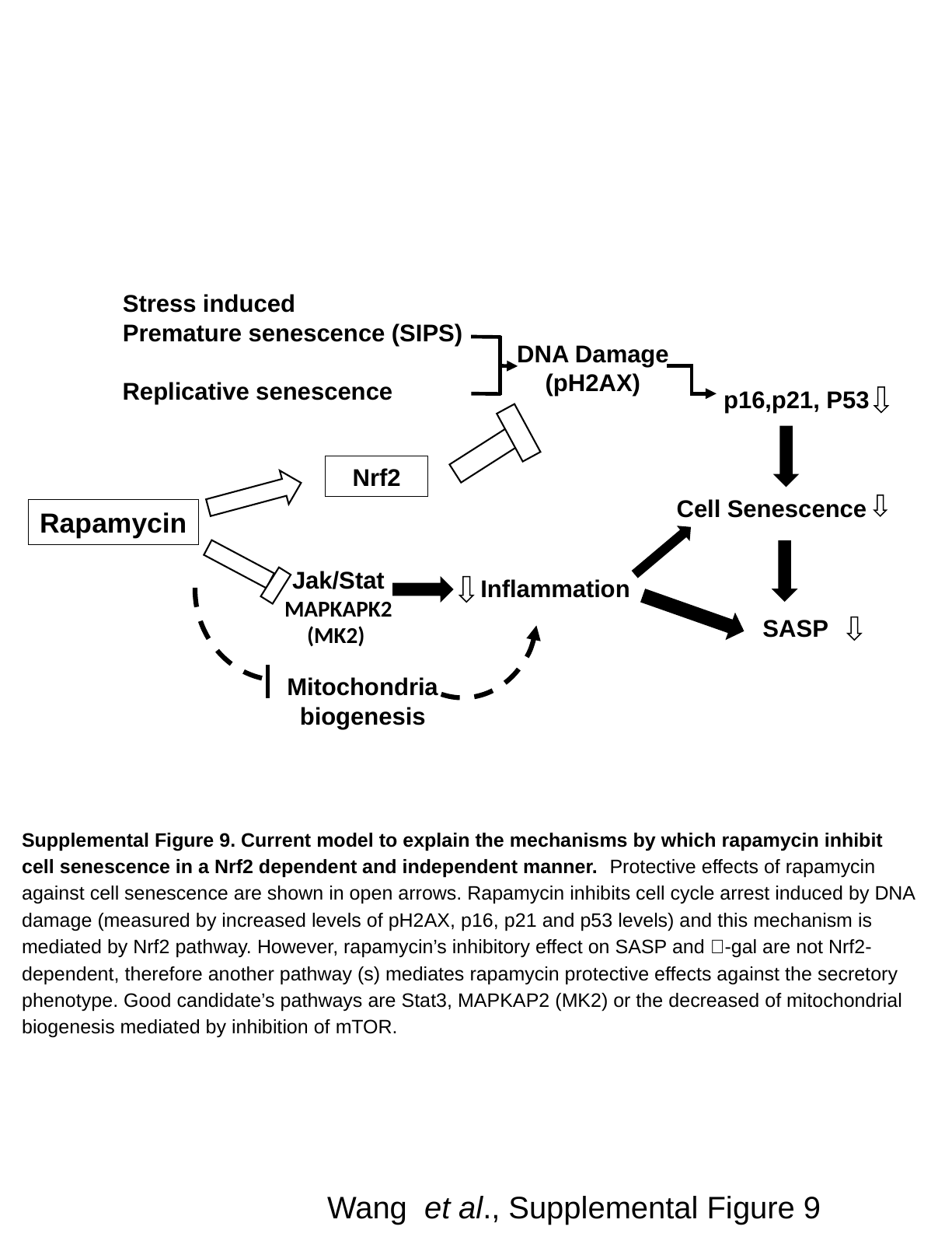

Stress induced
Premature senescence (SIPS)
DNA Damage
(pH2AX)
Replicative senescence
p16,p21, P53
Nrf2
Cell Senescence
Rapamycin
Jak/Stat
MAPKAPK2 (MK2)
Inflammation
SASP
Mitochondria
biogenesis
Supplemental Figure 9. Current model to explain the mechanisms by which rapamycin inhibit cell senescence in a Nrf2 dependent and independent manner. Protective effects of rapamycin against cell senescence are shown in open arrows. Rapamycin inhibits cell cycle arrest induced by DNA damage (measured by increased levels of pH2AX, p16, p21 and p53 levels) and this mechanism is mediated by Nrf2 pathway. However, rapamycin’s inhibitory effect on SASP and β-gal are not Nrf2-dependent, therefore another pathway (s) mediates rapamycin protective effects against the secretory phenotype. Good candidate’s pathways are Stat3, MAPKAP2 (MK2) or the decreased of mitochondrial biogenesis mediated by inhibition of mTOR.
Wang et al., Supplemental Figure 9
