# Supplementary material for: Rapamycin inhibits the secretory phenotype of senescent cells by a Nrf2‐independent mechanism
Source: Aging Cell. 2017 Mar 31;16(3):564–74. doi: 10.1111/acel.12587 (PMC5418203; doi:10.1111/acel.12587)
Supplement: Supplementary file 2 — Appendix S1 Experimental procedure. [file ACEL-16-564-s002.docx]

Support data

**Experimental procedure**

**Cell culture and treatment**

Wild-type and Nrf2 knock-out mouse embryo fibroblasts (MEF) are from Dr. Masayuki, Yamamoto Kohoku University of Japan.

**Quantitative real time PCR**

The mRNA was extracted using the RNeasy kit (Qiagen, Valencia, CA). RNA was reverse-transcribed to cDNA using SuperScript® III First-Strand Synthesis SuperMix following manufacturer’s instruction (Invitrogen). Target mRNA levels were measured by qPCR and normalized to actin beta (actb). Forty cycles of PCR (95^o^C/10s, 58^o^C/20s, 72^o^C/20s) were run on an Lightcycle480 system (Roche), in 10µl total reaction volume containing cDNAs, SYBR Green I dye (Roche) and primer set. The amount of specific mRNA was quantified by determining the point at which the fluorescence accumulation entered the exponential phase (C_t_), and the C_t_ ratio of the target gene to actb was calculated for each sample. Primers for mouse species were as follows:

TNFα F: 5’AGCCCCCAGTCTGTATCCTT3’, R: 5’ CTCCCTTTGCAGAACTCAGG3’;

Vcam1 F: 5’GTGGTGCTGTGACAATGACC3’, R: 5’ACGTCAGAACAACCGAATCC3’;

MMP3 F: 5’TGGAGATGCTCACTTTGACG3’, R: 5’GCCTTGGCTGAGTGGTAGAG3’;

MCP2 (CCL8) F: 5’ACGCTAGCCTTCACTCCAAA3’, R: 5’ AGCAGGTGACTGGAGCCTTA3’;

IL1β F: 5’ GACCTTCCAGGATGAGGACA3’, R: 5’ AGGCCACAGGTATTTTGTCG3’;

IL6 F: 5’ CCGGAGAGGAGACTTCACAG3’, R: 5’ TCCACGATTTCCCAGAGAAC3’;

CXCL1 F: 5’AGACTGCTCTGATGGCACCT3’, R: 5’TGCACTTCTTTTCGCACAAC3’;

CCL9 F: 5’AGTGGTCTGTGGGACTTTGG3’, R: 5’CAGACCTGTGGCTGCATAGA3’;

p16 F: 5’GAAGCCGGGGTTTCGCCCAA3’, R: 5’GCACCGGGCGGGAGAAGGTA3’

p21 F: 5’ACATTCAGAGCCACAGGCACCA3’, R: 5’GCATCGCAATCACGGCGCAA3’

NQO1: F: 5’TTCTCTGGCCGATTCAGAGT3’, R: 5’GGCTGCTTGGAGCAAAATAG3’ GSTya: F: 5’CGCCACCAAATATGACCTCT3’, R: 5’CCTGTTGCCCACAAGGTAGT3’. Primes for human species were as follows:

IL6 F: 5’ TACCCCCAGGAGAAGATTCC3’ ; R : 5’TTTTCTGCCAGTGCCTCTTT3’

IL1β F: 5’ CAGCCAATCTTCATTGCTCA3’; R: 5’GCATCTTCCTCAGCTTGTCC3’

Vcam1 F: 5’AAGATGGTCGTGATCCTTGG3’, R: 5’GGTGCTGCAAGTCAATGAGA3’

**Western blotting**

20µg protein were subjected by SDS-PAGE and processed by immunoblotting using antibody to monoclonal rabbit anti-NRF2 (D129C, 1:500 dilutions, Cell signaling, #12721), rabbit anti-p16INK4A (1:1000 dilution, Proteintech #100883-1-AP), rabbit anti-p62/SQSTM1 (1:1000, Cell signaling, #5114), monoclonal rabbit anti-LC3B (D11, 1:1000, Cell signaling, #3868), goat anti-keap1 (E20, 1:500 dilution, sc-15246), mouse anti-p21 (1:500 dilution, BD Bioscience, #556430), rabbit anti-p-Histone H2A.X Antibody (Ser 139, 1:250 dilution, Santa Cruz sc-101696), goat anti-NQO1 (1;500 dilution, 1:500 dilution, Santa Cruz sc-16494), monoclonal rabbit anti-ATF4 (D4B8,1:1000 dilution, #11815), mouse anti-phospho-Stat3 (Try 705, 1:500 dilution, Santa Cruz, sc-8057), monoclonal rabbit anti-stat3 (79D7, 1:1000 dilution, cell signaling #4904), rabbit anti-NFκB-p105/p50 (1:1000, Cell signaling, #3035), rabbit anti-NFκB-p65 (1:1000, Cell signaling, #3034), monoclonal mouse anti-action (C4, 1:8000 dilution, MP Biomedicals, # 08691). The proteins were visualized by Western Lightning^TM^ chemiluminscence Regent Plus (PerkinElmer Life Sciences, Boston MA, USA) and quantified using Rio-Rad Image System (USA).

**Overexpression with NFR2**

Plasmid cDNA of human NFE2L2 with Myc tag was from AddGene donated by Dr. Yue Xiong.

**Cytokine antibody array**

Cell conditioned media from wild type and Nrf2 null MEF cells treated 6 days with rapamycin or DMSO were collected after 2 days in serum free media. Proinflammatory factor levels were measured by using mouse cytokine antibody array (RayBio® C2000) according to the manufacturer's instructions. Briefly, after incubated with blocking buffer at room temperature for 30min, antibody array membranes were incubated with 1ml conditioned media at 4 ^o^C overnight, then washed with wash buffer I and II respectively for 3 times, and incubated with Biotinylated antibody cocktail at 4 ^o^C overnight. After repeating wash, membranes were incubated with HRP-streptavidin at 4 overnight. After repeating wash, cytokine levels were visualized by detection buffer and quantified using Rio-Rad Image System (USA). The intensities of signals were quantified, normalized to positive control, and calculated as log_2_-fold changes relative to untreated wild type MEF (baseline).

**Cell proliferation BrdU incorporation assay.**

MEF cell proliferation was measured with BrdU (5-bromo-2'-deoxyuridine) ELISA (colorimetric) and immunocytochemistry staining. For ELISA assay, 2*10^4^ cell cells were seed on 96-well plate. For Immunocytochemistry staining, 10^5^ cells were seed on poly-L-lysine coated coverslips in 12-well plates. Nrf2 re-expression, SISP induction, and pre- and treatment with Rapa were performed as described above. After incubated with Rapa for 24 H, cells continue growing in IMDM media without Rapa for 48H, then incubated with BrdU (10uM) for 2H. ELISA was performed following the manufacturer’s instructions (Abcam, ab126556). BrdU incorporation rate was expressed as OD value read at 450/595 nm dual wavelength.

For immunocytochemistry staining, cells were fixed with 5% buffered formalin phosphate, pH 7.2 for 10 min, washed with PBS, and incubated with methanol for 15 min. After treatment with 2.1% citric acid solution containing 0.5% Tween 20 for 10 min, cells were incubated with 1% BSA in PBS for 1 h. Rat anti- Brdu antibody (Bio-Rad, BU1/75, ICR1,1:300 dilution) was incubated overnight at 4 °C, and after washing with PBS, Anti-rat IgG (H+L) antibody (Alexa Fluor® 488 fluorescent dye conjugate, Cell signaling #4416) was applied for 1 h at room temperature. ProLong® Gold Antifade with DAPI (Life Technologies) was applied and cells were examined by fluorescence microscope. Cells were counted in 10 fields under the microscope with 200X magnification and expressed as a percentage of positive cells.
